# Adiponectin controls the apoptosis and the expression of tight junction proteins in brain endothelial cells through AdipoR1 under beta amyloid toxicity

**DOI:** 10.1038/cddis.2017.491

**Published:** 2017-10-12

**Authors:** Juhyun Song, Seong-Min Choi, Daniel J Whitcomb, Byeong C Kim

**Affiliations:** 1 Department of Biomedical Sciences, Center for Creative Biomedical Scientists at Chonnam National University, Gwangju 61469, South Korea; 2 Department of Anatomy, Chonnam National University Medical School, Gwangju 61469, South Korea; 3 Department of Neurology, Chonnam National University Medical School, Gwangju 61469, South Korea; 4 Henry Wellcome Laboratories for Integrative Neuroscience and Endocrinology, School of Clinical Sciences, Faculty of Healthy Sciences, University of Bristol, Whitson Street, Bristol BS1 3NY, UK

## Abstract

Alzheimer’s disease (AD) is the most common neurodegenerative disease, characterized by excessive beta amyloid (A*β*) deposition in brain, leading to blood–brain barrier (BBB) disruption. The mechanisms of BBB disruption in AD are still unclear, despite considerable research. The adipokine adiponectin is known to regulate various metabolic functions and reduce inflammation. Though adiponectin receptors have been reported in the brain, its role in the central nervous system has not been fully characterized. In the present study, we investigate whether adiponectin contributes to the tight junction integrity and cell death of brain endothelial cells under A*β*-induced toxicity conditions. We measured the expression of adiponectin receptors (AdipoR1 and AdipoR2) and the alteration of tight junction proteins in *in vivo* 5xFAD mouse brain. Moreover, we examined the production of reactive oxygen species (ROS) and the loss of tight junction proteins such as Claudin 5, ZO-1, and inflammatory signaling in *in vitro* brain endothelial cells (bEnd.3 cells) under A*β* toxicity. Our results showed that Acrp30 (a globular form of adiponectin) reduces the expression of proinflammatory cytokines and the expression of RAGE as A*β* transporters into brain. Moreover, we found that Acrp 30 attenuated the apoptosis and the tight junction disruption through AdipoR1-mediated NF-*κ*B pathway in A*β*-exposed bEnd.3 cells. Thus, we suggest that adiponectin is an attractive therapeutic target for treating BBB breakdown in AD brain.

Alzheimer’s disease (AD) is a neurodegenerative disease characterized by toxic plaques that consist of beta amyloid (A*β*) peptides generated from amyloid precursor protein (APP).^1, 2, 3^ The excessive accumulation of these plaques in the brain impairs synaptic function and leads to neuronal death, ultimately manifesting in memory dysfunction.^1, 4^ A*β* has been known to trigger a synaptopathy of reactive oxygen species (ROS) production, neuronal cell death, glia activation, and tau hyperphosphorylation.^5^ In addition to directly effecting neurons, A*β* is also known to be deposited on the walls of blood vessels and induce inflammation in endothelial cells.^6, 7^ A critical consequence of this is the disruption of the blood–brain barrier (BBB) integrity^8, 9^ through ROS production and secretion of pro-inflammatory cytokines.^10^ BBB comprises several cells including brain endothelial cells, interconnected by tight junctions consisting of the junctional adhesion molecule 1 (JAM-1), zona occludens 1 (ZO-1), occludin, and claudin.^11, 12^ In AD, BBB is damaged by A*β* accumulation;^13, 14^ its structure is changed by the disruption of tight junction proteins and the permeability of BBB is elevated during the progress of disease.^15, 16^ For these reasons, recent researchers have focused on understanding the BBB disruption-related mechanisms under A*β* accumulation in order to uncover effective solutions for alleviating AD pathology,^17, 18, 19^ though a decisive target remains to be determined.

Adiponectin is a 244 amino acid polypeptide adipokine encoded by the ADIPOQ gene.^20^ It binds to two receptors (AdipoR1 and AdipoR2),^21, 22^ which exist in the brain as well as other organs throughout the body.^23, 24^ Adiponectin is known to play key roles as an insulin sensitizer and an anti-inflammatory regulator, in addition to the regulation of glucose metabolism and fatty acid breakdown.^25, 26^ In the central nervous system, previous reports suggest that adiponectin modulates memory function and has a protective effect on neurons and neural stem cells against stress condition.^27, 28^ One study showed that serum adiponectin levels were lower in APP transgenic mice compared with control mice and outlined an association with inflammation and cognitive dysfunction in AD.^29^ Moreover, adiponectin reduces the secretion of interleukin-6 (IL-6) from brain endothelial cells in response to oxidative stress, modulating BBB function.^30^ Judging from previous evidences, adiponectin has the potential to play a cellular protective role in brain endothelial cells under A*β*-induced oxidative stress and attenuate the BBB disruption caused by A*β* accumulation in AD brain.

In the present study, we investigated whether adiponectin contributes to the apoptosis of brain endothelial cells and the loss of tight junction under A*β* toxicity condition. Our findings suggest that adiponectin may protect BBB disruption in the AD brain by alleviating the damage of brain endothelial cells caused by A*β* toxicity.

## Results

### The expression of adiponectin receptors was reduced in 5xFAD mouse brain

**Figure 1:**
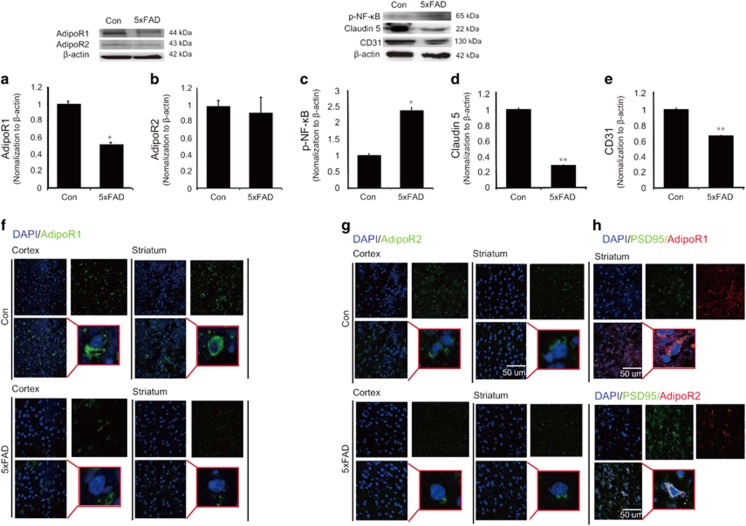
The expression of adiponectin receptors and the activation of NF-*κ*B and the decreased Claudin 5 protein in 5xFAD mouse brain. Western blotting showed the protein level of AdipoR1 (**a**) and AdipoR2 (**b**) in 5xFAD mouse brain. (**a**) The protein level of AdipoR1 was significantly reduced in 5xFAD mouse brain compared to the control mouse brain. (**b**) The protein level of AdipoR2 was slightly reduced in 5xFAD mouse brain compared to the control mouse brain. (**c**) Western blotting showed the protein level of p-NF-*κ*B in mouse brain. The protein level of p-NF-*κ*B was significantly increased in 5xFAD mouse brain compared to the control mouse brain. (**d**) Western blotting showed the protein level of Claudin 5 in mouse brain. The protein level of Claudin 5 was significantly reduced in 5xFAD mouse brain in comparison with the control mouse brain. (**e**) The protein level of CD31 was slightly reduced in 5xFAD mouse brain compared to the control mouse brain. Data are expressed as mean±S.E.M., and each experiment conducted three repeats per conditions. *β*-actin was used as control. Differences were considered significant at *P<0.05, **P<0.01. Images showed the expression of AdipoR1 (Green) (**f**) and AdipoR2 (Green) (**g**). 5xFAD mouse showed the less expression of AdipoR1 (**f**) and AdipoR2 (**g**). (**h**) PSD95-positive cells matched with the AdipoR1 and AdipoR2 staining cell in brain. Con: control normal mouse, 5xFAD: 5xFAD mouse, Scale bar: 50 *μ*m, 4',6-diamidino-2-phenylindole (DAPI): blue, AdipoR1: adiponectin receptor 1 (Green), AdipoR2: adiponectin receptor 2, p-NF-*κ*B: phosphorylation of NF-kB, PSD95: postsynaptic density protein 95

To examine the expression of adiponectin receptors (AdipoR1 and AdipoR2) in 5xFAD mouse brain, we measured the expression of adiponectin receptors through western blotting (Figures 1a and b) and immunostaining (Figures 1f and g). In 5xFAD mouse brain, the protein level of AdipoR1 was significantly reduced compared with the control mouse brain (Con) (Figure 1a). The protein level of AdipoR2 also showed a slight, albeit non-significant decrease of protein level in 5xFAD mouse brain in comparison with the normal mouse brain (Figure 1b). The immunostaining images showed considerable reduction of AdipoR1 in 5xFAD mouse brain entorhinal cortex and striatum (Figure 1f). Figure 1g presents the reduction of AdipoR2 in 5xFAD mouse brain entorhinal cortex and striatum (Figure 1g). These data suggest that levels of adiponectin receptors (AdipoR1 and AdipoR2) are altered in 5xFAD mouse brain (Figures 1a). Figure 1h shows the PSD95 (postsynaptic protein95; considered as neuron) and AdipoR1, AdipoR2 colocalization in brain (Figure 1h). Based on our results of the colocalization of PSD95 and AdipoR1 or AdipoR2, we showed the expression of AdipoR1 and AdipoR2 in neuronal cells.

### The increase of NF-*κ*B phosphorylation and the loss of tight junction protein in 5xFAD mouse brain

To examine whether AD triggers the inflammatory signaling such as NF-*κ*B, we assess the alterations of NF-*κ*B phosphorylation (Figure 1c). Also, to check the loss of tight junction proteins (Figure 1d) that may occur as a result of AD pathology, we conducted western blotting analysis using Claudin 5 antibody by extracting the proteins in the 5xFAD mouse brain (Figures 1c and d). Figure 1e shows the protein level of CD31 as a vascular marker (Figure 1e). Western blotting data showed that the activation of NF-*κ*B was markedly increased in 5xFAD mouse brain in comparison with the control mouse brain (Figure 1c), suggestive of the activation of NF-*κ*B pathway in 5xFAD mouse brain (Figure 1c). Also, western blotting data suggested that Claudin 5 junction protein was considerably reduced in 5xFAD mouse brain (Figure 1d). This result demonstrates a relative loss of tight junction protein Claudin 5 in 5xFAD mouse brain compared with the control mouse brain (Figure 1d).

### The reduction of cell viability and the increase of nitrite oxide production in A*β*-treated brain endothelial cells

**Figure 2:**
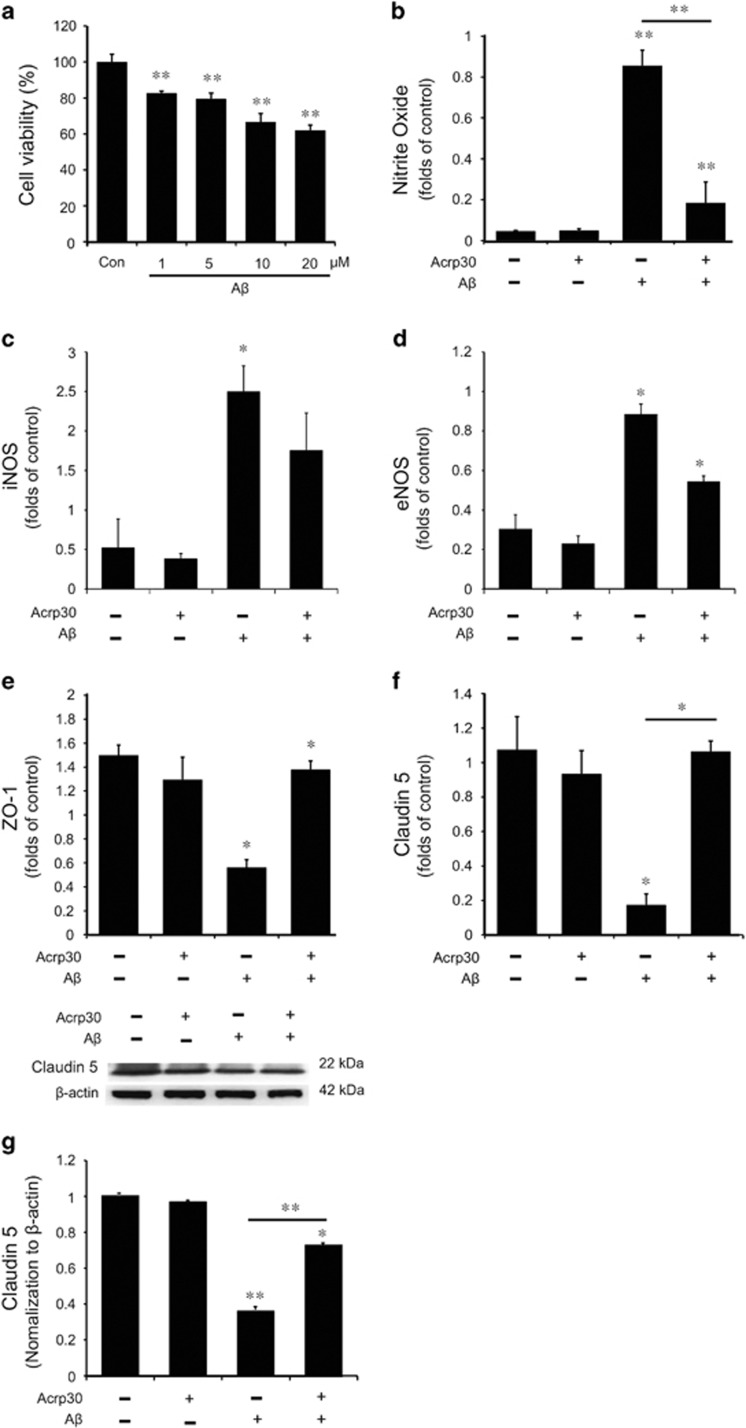
The measurement of nitric oxide production and tight junction protein in bEnd.3 cells under A*β* toxicity. (**a**)The cell viability in bEnd.3 cells under A*β*-induced toxicity was assessed by MTT assay. bEnd.3 cells were treated with A*β* at 1, 5, 10, 20 *μ*M for 24 h. The cell viabilities in bEnd.3 cells treated with A*β* 10 *μ*M and 20 *μ*M concentration were showed below 70% compared to that in control group (only DMSO). The value was calculated as 100% of control (only DMSO). (**b**) The production of nitrite was measured by Griess reagent assay. The production of nitrite was increased in A*β*-treated bEnd.3 cells, and pre-treatment of Acrp 30 reversed the A*β*-induced increase of nitrite production. (**c**) The mRNA level of iNOS was checked with reverse transcription PCR. The mRNA of iNOS was increased in bEnd.3 cells under A*β*-induced toxicity. (**d**) The mRNA level of eNOS was measured with reverse transcription PCR. The mRNA of eNOS was considerably increased in bEnd.3 cells under A*β*-induced toxicity. Pre-treatment of Acrp30 reversed the A*β*-induced increase of iNOS mRNA level in bEnd.3 cells. The mRNA levels of Claudin 5 (**f**) and ZO-1 (**e**) were measured with quantitative real-time PCR. The mRNA levels of Claudin 5 and ZO-1 were reduced in bEnd.3 cells under A*β*-induced toxicity. (**e**) Pre-treatment of Acrp30 reversed the A*β*-induced decrease of ZO-1 mRNA levels in bEnd.3 cells. (**f**) quantitative real-time PCR also revealed that A*β*-induced decrease of Claudin 5 mRNA was reversed by pre-treatment of Acrp30 in bEnd.3 cells. (**g**) Western blotting data revealed that A*β*-induced decrease of Claudin 5 protein level was reversed by pre-treatment of Acrp30 in bEnd.3 cells. Differences were considered significant at *P<0.05, **P<0.001. Data are expressed as mean±S.E.M. GAPDH gene and *β*-actin were used as control. Con: only DMSO, Acrp30: Acrp 30 10 *μ*g/ml treatment for 24 h, A*β*: A*β* 20 *μ*M treatment for 24 h, iNOS: inducible nitric oxide synthase

To investigate the toxicity of A*β* in the brain endothelial cells, we measured cell viability in bEnd.3 cells by MTT assay (Figure 2a). The cell viability of brain endothelial cells was approximately 70% in 10 *μ*M A*β*-treated group and 60% in 20 *μ*M A*β*-treated group (Figure 2a). We treated 20 *μ*M A*β* for 24 h in bEnd.3 cells to study the effect of adiponectin in brain endothelial cells against A*β*-induced stress condition. When we treated 20 *μ*M A*β* for 24 h in bEnd.3 cells, we observed a marked increase of NO production in bEnd.3 cells. Pre-treatment of Acrp 30 (as an adiponectin globular form)^31^ 10 *μ*g/ml leads to the considerable decrease of NO production in bEnd.3 cells, despite A*β*-induced stress condition (Figure 2b). In addition, we checked the mRNA level of inducible nitric oxide synthases (iNOS) (as an inducer of NO production)^32^ (Figure 2c) and endothelial nitric oxide synthases (eNOS) by RT-PCR in A*β*-treated bEnd.3 cells (Figure 2d). The mRNA level of iNOS was increased in A*β*-treated bEnd.3 cells, and pre-treatment of Acrp 30 reversed A*β*-induced increase of iNOS mRNA level (Figure 2c). The mRNA level of eNOS was increased in A*β*-treated bEnd.3 cells, and pre-treatment of Acrp 30 inhibited A*β*-induced increase of eNOS mRNA level (Figure 2d).

### The decrease of tight junction proteins in A*β*-treated brain endothelial cells

To assess the alterations of tight junction proteins (Claudin 5 and ZO-1) in A*β*-treated brain endothelial cells, we conducted quantitative real-time PCR (Figures 2e and f) and western blotting (Figure 2g). Our results showed that the mRNA levels of Claudin 5 (Figure 2f) and ZO-1 (Figure 2e) were decreased in A*β*-treated bEnd.3 cells. The loss of Claudin 5 protein in A*β*-treated bEnd.3 cells was suppressed by Acrp 30 (10 *μ*g/ml) pre-treatment for 24 h (Figure 2g). These data suggest that Acrp 30 prevents the loss of tight junction proteins such as Claudin 5 and ZO-1 in A*β*-treated bEnd.3 cells (Figures 2e–g).

### Acrp30 rescues cell death and inhibits the production of ROS in bEnd.3 cells under A*β*-induced oxidative stress condition

**Figure 3:**
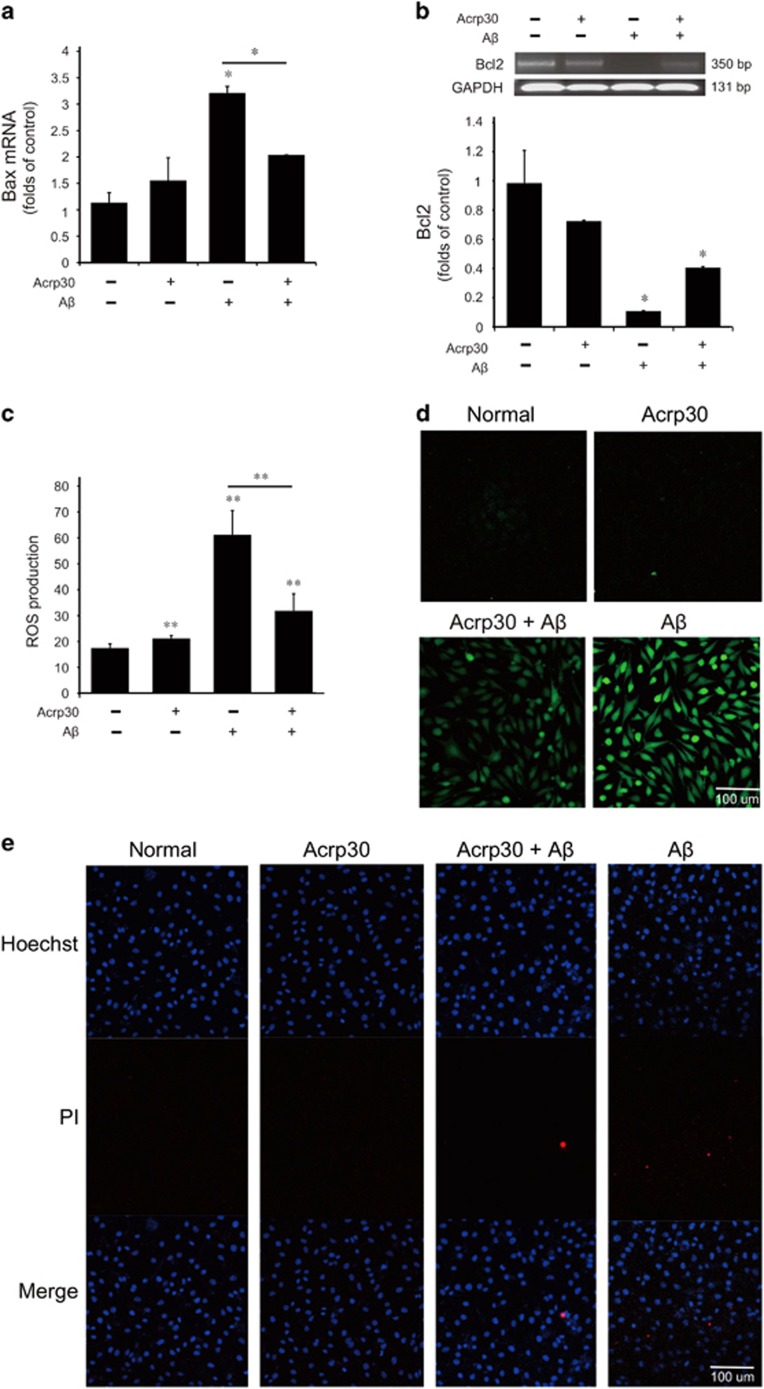
Adiponectin rescues cell death and inhibits the production of ROS under amyloid beta toxicity. The mRNA levels of Bax (**a**) were assessed with quantitative real-time PCR. Also, the mRNA levels of Bcl2 (**b**) were measured with reverse transcription PCR. A*β* treatment induced increase of Bax mRNA level and decrease of Bcl2 mRNA level in bEnd.3 cells, and pre-treatment of Acrp30 reversed those changes. (**c**,**d**) The production of ROS was measured using DCF-DA reagent. Differences were considered significant at *P<0.05, **P<0.001. Data are expressed as mean±S.E.M. GAPDH was used as control gene. A*β*-treated bEnd.3 cells showed increase of ROS production, and pre-treatment of Acrp30 reversed A*β*-induced increase of ROS production in bEnd.3 cells. (**e**) PI-positive cells (red color) were considered as the dead cells. Scale bar: 100 *μ*m, ROS: green, Acrp30: Acrp 30 10 *μ*g/ml treatment for 24 h; A*β*: A*β* 20 *μ*M treatment for 24 h, Hoechst: blue color, propidium iodide (PI): red color

To examine whether adiponectin contributes to the cell death and the production of ROS in A*β*-exposed brain endothelial cells, we checked the mRNA levels of Bax (Figure 3a) by quantitative real-time PCR and Bcl2 (Figure 3b) by reverse transcription PCR and the production of ROS by 2′,7′-dichlorofluorescin diacetate (DCF-DA) assay in bEnd.3 cells (Figures 3c and d). To confirm the alteration of cell death by adiponectin, we conducted Hoechst/PI staining (Figure 3e). We observed that PI-positive cells (death cells) were reduced by adiponectin treatment under A*β* toxicity (Figure 3e). Our results indicated that pre-treatment of Acrp 30 reversed A*β*-induced increase of Bax expression, reduction of Bcl2 expression, and increase of ROS production in bEnd.3 cells (Figure 3).

### The expression of pro-inflammatory cytokines in bEnd.3 cells under A*β* toxicity

**Figure 4:**
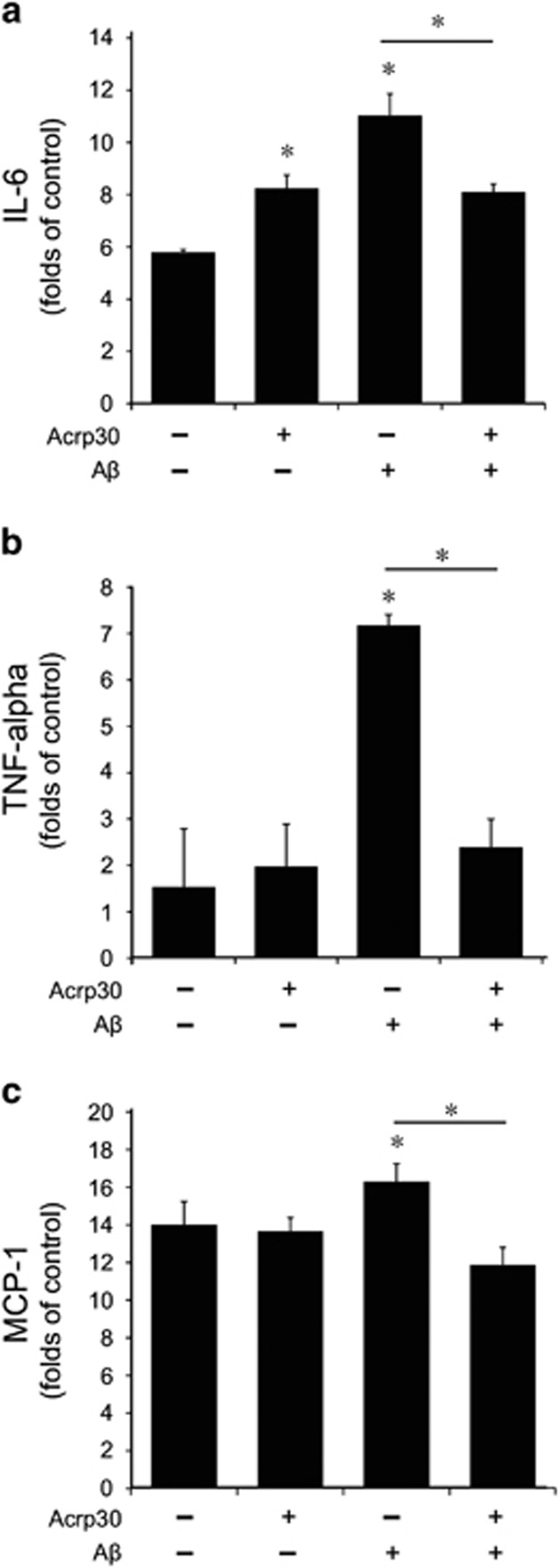
The expression of pro-inflammatory cytokines in bEND3 cells. The mRNA levels of IL-6, TNF-*α*, MCP-1 were detected by quantitative real-time PCR. A*β* treatment in bEnd.3 cells induced increase of IL-6 (**a**), TNF- *α* (**b**), and MCP-1 (**c**) mRNA levels. Pre-treatment of Acrp 30 reversed A*β*-induced increases of each mRNA levels in bEnd.3 cells (**a**–**c**). Data are expressed as mean±S.E.M. GAPDH was used as control gene. Differences were considered significant at *P<0.05. Acrp30: Acrp 30 10 *μ*g/ml treatment for 24 h, A*β*: A*β* 20 *μ*M treatment for 24 h

To test the expression of inflammatory cytokines in A*β*-treated brain endothelial cells, we checked the mRNA level of IL-6, tumor necrosis factor *α* (TNF-*α*), and monocyte chemoattractant protein-1 (MCP-1) in bEnd.3 cells by quantitative real-time PCR (Figure 4). We observed that the expression of IL-6 was slightly increased by Acrp 30 treatment (Figure 4a). A*β* treatment triggered the expression of pro-inflammatory cytokine IL-6 (Figure 4a), TNF-*α* (Figure 4b), and MCP-1 (Figure 4c) in bEnd.3 cells, whereas pre-treatment of Acrp 30 reduced the increased expression of IL-6 (Figure 4a), TNF-*α* (Figure 4b), and MCP-1 (Figure 4c) in A*β*-exposed bEnd.3 cells.

### A*β* leads to the decreased expression of adiponectin receptor in brain endothelial cells

**Figure 5:**
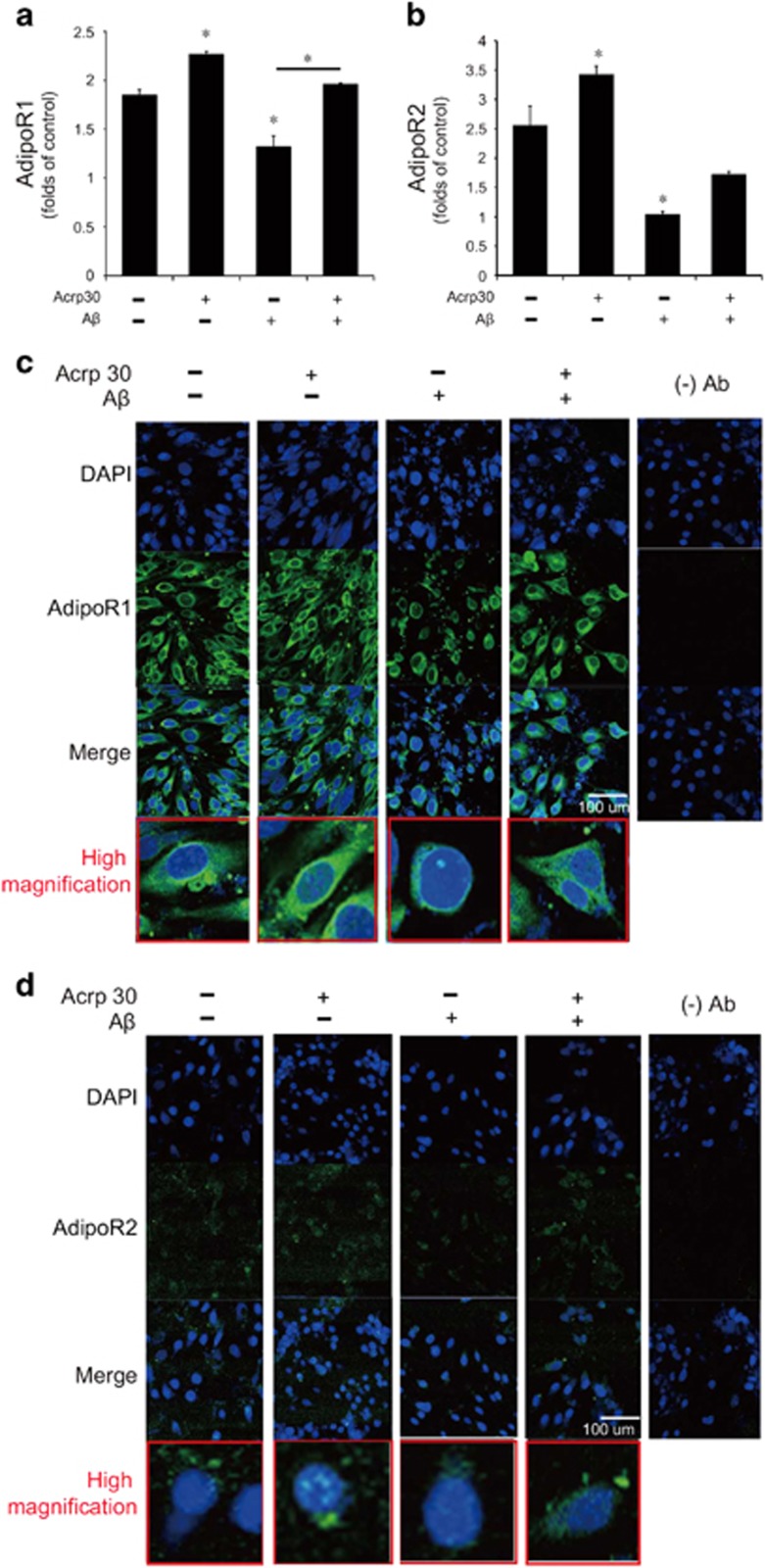
The expression of adiponectin receptors in bEnd.3 cells under amyloid beta toxicity. The mRNA level of AdipoR1 (**a**) and AdipoR2 (**b**) were checked by reverse transcription PCR. The mRNA level of AdipoR1 in bEnd.3 cells was reduced by A*β* treatment, and pre-treatment of Acrp 30 reversed A*β*-induced decrease of AdipoR1 in bEnd.3 cells (**a**,**c**). The mRNA level of AdipoR2 in bEnd.3 cells was also reduced by A*β* treatment, whereas pre-treatment of Acrp 30 did not reverse A*β*-induced decrease of AdipoR2 in bEnd.3 cells (**b**). Data are expressed as mean±S.E.M. GAPDH was used as control gene. Differences were considered significant at *P<0.05, **p<0.001. The immunostaining images showed the expression of AdipoR1 (**c**) and AdipoR2 (**d**) in bEnd.3 cells. Scale bar: 100 *μ*m, 4',6-diamidino-2-phenylindole (DAPI): blue, AdipoR1: green, AdipoR2: green, Acrp30: Acrp 30 10 *μ*g/ml treatment for 24 h, A*β*: A*β* 20 *μ*M treatment for 24 h. Con: no treatment group, (−)Ab: IgG control

To confirm the change of expression of adiponectin receptors in brain endothelial cells under A*β* toxicity, we checked the expression of AdipoR1 and AdipoR2 by quantitative real-time PCR (Figures 5a and b) and immunocytochemistry (Figures 5c and d). A*β* treatment resulted in the reduction of the mRNA levels of AdipoR1 and AdipoR2 in bEnd.3 cells (Figures 5a and b). In Figures 5c and d, immunostaining data shows the expression of AdipoR1 and AdipoR2 in bEND.3 cells (Figures 5c and d). Pre-treatment of Acrp 30 reversed A*β*-induced decrease of AdipoR1, but not of AdipoR2 in bEnd.3 cells (Figures 5a–d).

### Acrp30 changes the expression of RAGE and LRP-1 in bEnd.3 cells under A*β* toxicity

**Figure 6:**
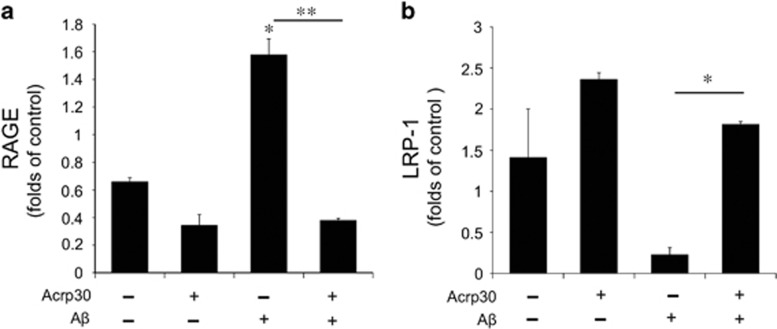
The mRNA levels of RAGE and LRP-1 in bEnd.3 cells under amyloid beta toxicity. The mRNA level of RAGE and LRP-1 were measured by quantitative real-time PCR. The mRNA level of RAGE (**a**) was increased and that of LRP-1 (**b**) was decreased in bEnd.3 cells by A*β* treatment. Pre-treatment of Acrp 30 reversed A*β*-induced changes of RAGE (**a**) and LRP-1 (**b**) mRNA levels in bEnd.3 cells. Data are expressed as mean±S.E.M. GAPDH was used as control gene. Differences were considered significant at *P<0.05, **P<0.001. Acrp30: Acrp 30 10 *μ*g/ml treatment for 24 h, A*β*: A*β* 20 *μ*M treatment for 24 h

To check the expression of receptor for advanced-glycation end products (RAGE) and low-density lipoprotein receptor-related protein 1 (LRP-1) in brain endothelial cells under A*β* toxicity, we measured the mRNA levels of RAGE and LRP-1 in A*β*-treated bEnd.3 cells by quantitative real-time PCR (Figure 6). A*β* triggered the increase of RAGE mRNA level in bEnd.3 cells, while Acrp 30 reversed A*β*-induced increase of RAGE mRNA level (Figure 6a). A*β* triggered the decrease of LRP-1 mRNA level in bEnd.3 cells, and Acrp 30 reversed A*β*-induced decrease of LRP-1 mRNA level (Figure 6b).

### Acrp 30 protects the tight junction integrity and attenuates the inflammatory responses through AdipoR1

**Figure 7:**
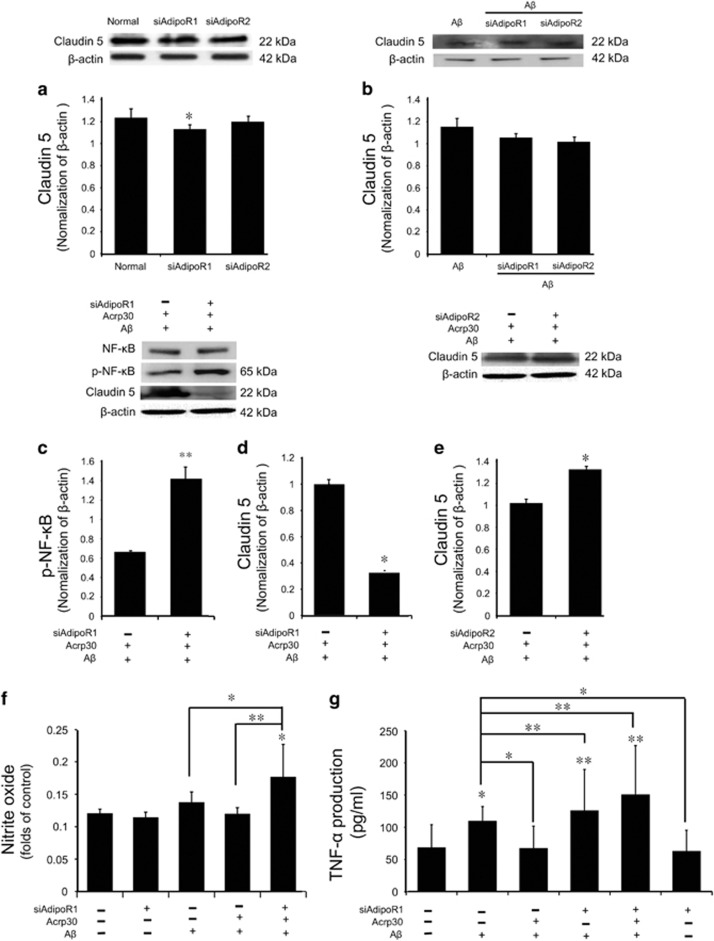
The change of inflammatory response and the tight junction proteins by inhibiting Adiponectin receptor under amyloid beta toxicity. The protein level of claudin5 was assessed by western blotting in AdipoR1, AdipoR2 knockout condition (**a**,**b**). Panel **b** shows the protein level of Claudin 5 in AdipoR1, AdipoR2 knockout condition under A*β* treatment (**b**). The protein level of p-NF-*κ*B (**c**) and Claudin 5 (**d**) were detected by western blotting. (**c**) The protein level of p-NF-*κ*B was increased in A*β*-treated bEnd.3 cells with Acrp 30 pre-treatment under AdipoR1 knockdown. (**d**) The protein level of Claudin 5 was markedly decreased in A*β*-treated bEnd.3 cells with Acrp 30 pre-treatment under AdipoR1 knockdown. (**e**)The protein level of Claudin 5 was not decreased in A*β*-treated bEnd.3 cells with Acrp 30 pre-treatment under AdipoR2 knockdown. (**f**) The production of NO was checked by Griess reagent assay. The production of NO was increased in A*β*-treated bEnd.3 cells with Acrp 30 pre-treatment under AdipoR1 knockdown. (**g**) The production of TNF-*α* was checked by ELISA assay. The production of TNF-*α* was increased in A*β*-treated bEnd.3 cells with Acrp 30 pre-treatment under AdipoR1 knockdown. Data are expressed as mean±S.E.M. *β*-actin was used as control. Differences were considered significant at *P<0.05, **P<0.001.Acrp30: Acrp 30 10 *μ*g/ml treatment for 24 h, A*β*: A*β* 20 *μ*M treatment for 24 h, siAdipoR1: silencing of AdipoR1

**Figure 8:**
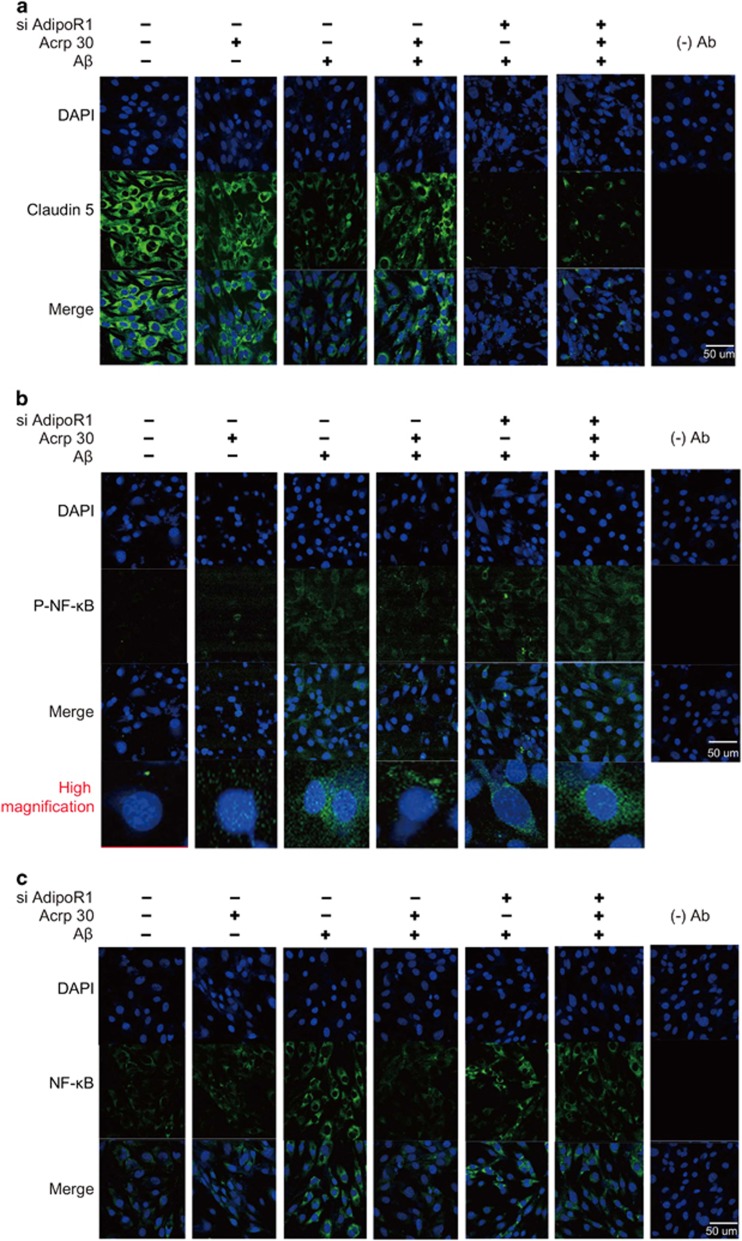
The alteration of Claudin 5 and phosphorylated NF-*κ*B protein’s expression by inhibiting AdipoR1. (**a**) The expression of Claudin 5 was assessed by immunocytochemistry. Pre-treatment of Acrp 30 did not reverse the A*β*-induced decrease of Claudin 5 expression in bEnd.3 cells in the presence of AdipoR1 knockdown using siRNA AdipoR1. (**b**) The expression of p-NF-*κ*B was checked by immunocytochemistry. Pre-treatment of Acrp 30 did not reverse the A*β*-induced increase of p-NF-*κ*B expression in bEnd.3 cells in the presence of AdipoR1 knockdown using siRNA AdipoR1. (**c**) The expression of NF-*κ*B was checked by immunocytochemistry in bEnd.3 cells in the presence of AdipoR1 knockdown using siRNA AdipoR1.Acrp30: Acrp 30 10 *μ*g/ml treatment for 24 h, A*β*: A*β* 20 *μ*M treatment for 24 h, siAdipoR1: silencing of AdipoR1, Scale bar: 50 *μ*m, 4',6-diamidino-2-phenylindole (DAPI): blue, Claudin 5: green, NF-kB: green, p-NF-kB: green, (−) Ab: IgG control

First, we checked the protein level of Claudin5 in AdipoR1 and AdipoR2 knockout condition (Figures 7a and b). We used appropriate non-silencing control siRNA and confirmed the efficiency of the siRNA knockdown in this study. We confirmed almost the same protein level of claudin5 in AdipoR1 and AdipoR2 knockout condition (Figure 7a). Also, we measured the protein level of Claudin 5 in AdipoR1 and AdipoR2 knockout cells under A*β* treatment (Figure 7b). We found that the protein level of Claudin 5 was not largely changed in AdipoR1 and AdipoR2 knockout condition under A*β* treatment condition (Figure 7b). To assess whether adiponectin boosts NF-*κ*B signaling (known as the inflammatory pathway) in A*β*-exposed brain endothelial cells through AdipoR1, we used siRNA AdipoR1 for knock-down of AdipoR1 in brain endothelial cells (Figure 7c). We observed the activation of NF-*κ*B in AdipoR1 knockdown group despite pre-treatment of Acrp 30 in A*β*-treated bEnd.3 cells (Figure 7c). In addition, to investigate whether adiponectin influences tight junction integrity through AdipoR1, we used siRNA AdipoR1 for knockdown of AdipoR1 in brain endothelial cells (Figure 7d). We observed loss of Claudin 5 by A*β* toxicity in AdipoR1 knockdown group despite Acrp 30 pre-treatment (Figure 7d). When we suppress the expression of AdipoR2 using siRNA AdipoR2, we found a little bit change of Claudin 5 protein level in brain endothelial cells under A*β* with Acrp30 treatment condition (Figure 7e). Moreover, we measured the production of NO by Griess reagent assay (Figure 7f) and the production of TNF-*α* by ELISA assay (Figure 7g) in bEnd.3 cells. Pre-treatment of Acrp 30 did not reverse A*β*-induced increase of nitric oxide in bEnd.3 cells in the presence of AdipoR1 knockdown (Figure 7f). Finally, we examined the expression of Claudin 5 (Figure 8a) and p-NF-*κ*B (Figure 8b), NF-*κ*B (Figure 8c) in bEnd.3 cells using immunocytochemisty (Figure 8). Images show that pre-treatment of Acrp 30 did not reverse the A*β*-induced changes of Claudin 5 expression and NF-*κ*B activation in bEnd.3 cell in the presence of AdipoR1 knockdown (Figure 8).

## Discussion

AD, a neurodegenerative disorder, is characterized by abnormal accumulation of A*β* and neurovascular dysfunction.^33^ The excessive A*β* deposition in brain endothelial cells aggravates the increase of BBB permeability by impairing BBB transport systems in the AD brain.^14, 34^ Adiponectin acts by binding with specific receptor AdipoR1 and AdipoR2,^21^ which exist in various organs including brain.^23, 24^ Considering that our results showed the AdipoR1 and AdipoR2 detected cells were neuronal cells in brain, we assume that the adiponectin receptors could be involved in the excitability of neurons^35^ and the suppression of neuronal damage against oxidative stress.^36^ Also, adiponectin has been reported that it is present in the cerebrospinal fluid (CSF) of rodents^37, 38^ and humans,^39, 40^ although the concentration of it in CSF is less than the concentration of it in plasma and is controversial whether or not it could cross the BBB.^30^

Recent studies suggested the protective potential of adiponectin on BBB breakdown in AD,^29, 30^ but the specific mechanisms remained to be fully characterized. In the present study, we found a reduction of AdipoR1 and AdipoR2 expression and the loss of tight junction protein Claudin 5 in models of AD pathology. These effects were concomitant with the activation of NF-*κ*B. Taken together, these data suggest that decreased expression of adiponectin receptors in the AD brain may be associated with BBB disruption and activation of the inflammatory response.

Excessive production of ROS can cause severe cell damage^41^ and can also increase vascular endothelial permeability and leukocyte adhesions.^16^ In addition, a large amount of ROS triggers loss of endothelial cell interactions^42^ and affects BBB integrity by disturbing tight junctions.^43, 44^ One study showed that adiponectin prevents the inflammation of vascular endothelial cells by reducing the secretion of ROS.^45^ Considering previous results and our *in vitro* results, we suggest that adiponectin may suppress A*β* toxicity-induced inflammation and BBB disruption by reducing the production of ROS in brain endothelial cells.

Previous study demonstrated that A*β* increases the expression of iNOS gene.^46^ iNOS produces NO and triggers the inflammatory response.^47^ Excessive production of NO in endothelial cells promotes pro-inflammatory signaling and the process of APP.^48, 49^ Adiponectin has been known to control the production of cytokines by regulating the expression of eNOS.^50^ In addition, adiponectin inhibits fibroblast migration through AdipoR1-AMPK-iNOS pathway in inflammatory condition.^51^ Based on our results, we infer that adiponectin contributes to the expression of cytokines and inflammation signaling by blocking the production of NO against A*β*-induced toxicity. Several studies demonstrated that adiponectin reduces the production of IL-6,^30^ IL-8, vascular endothelial growth factor and matrix metalloproteinases (MMPs) in endothelial cells.^52^ In addition, the overexpression of adiponectin receptors promotes the anti-inflammatory response in vascular endothelial cells.^53^ Also, adiponectin inhibits vascular endothelial hyperpermeability through cAMP/PKA signaling.^54^ Regarding our results, we suggest that adiponectin reduces the expression of pro-inflammatory cytokines including IL-6, TNF-*α* and MCP-1 in brain endothelial cells under A*β*-induced oxidative stress conditions.

In AD, the accumulation of A*β* in endothelial vessel walls leads to endothelial dysfunction^55^ and impaired BBB integrity.^56^ The loss of tight junction proteins such as occludin, ZO-1, and Claudin 5 by A*β* aggravates the increase of barrier permeability and apoptosis of vascular endothelial cells.^57, 58^ The alteration of tight junction and ROS production causes BBB leakage in AD brains.^59^ Recent study reported that the activation of NF-*κ*B by A*β* accumulation disrupts the integrity of BBB by decreasing Claudin 5 and increasing receptor for RAGE.^60^ In the present study, we found that adiponectin suppresses the loss of tight junction proteins and the increase of RAGE expression in brain endothelial cells under A*β*-induced toxicity.

In the AD brain, A*β* could be transported into the brain across the BBB and it is regulated by the BBB receptors and transporters.^61, 62^ A*β* accumulation in AD brain results from decreased clearance from BBB and increase of uptake from the circulatory system.^61, 62^ RAGE transports A*β* from the circulation into the brain,^63, 64^ whereas LRP-1 is related with rapid clearance of A*β* from the brain to blood.^65, 66, 67, 68^ The dysfunction of LRP-1 weakens the ability of BBB to clearing A*β*.^67^ Several clinical studies have demonstrated that the onset and procession of AD is associated with low LRP-1 levels and high RAGE levels, leading to the accumulation of A*β* peptides in the brain parenchyma.^69, 70^ One clinical study has observed that the expression of LRP-1 is reduced and the expression of RAGE is increased in AD patients.^71^ According to the current study, 5xFAD mice have decreased levels of LRP-1 receptor and lower levels of A*β* in plasma, with an increase of A*β* in the brain.^72^ A*β* oligomers are known to cause upregulation of RAGE expression in endothelial cells.^73^ This may have important consequences given that RAGE promotes the expression of MMP-2 related with permeability.^74, 75^ One study has reported that A*β* triggers the increase of permeability and the disruption of tight junction protein ZO-1 and the secretion of MMP in brain endothelial cells by interacting with RAGE.^15, 34^ In recent years, an inverse relationship was found to exist between the expression of adiponectin and RAGE.^76, 77^ Given that our results showed low mRNA level of RAGE and high mRNA level of LRP-1 in A*β*-exposed bEnd.3 cells by Acrp 30 treatment, we assume that adiponectin contributes to the transportation of A*β* into brain and the clearance of A*β* by enhancing the expression of LRP-1 and suppressing the level of RAGE in brain endothelial cells. Even though we did not find a direct mechanistic connection between adiponectin and A*β*-RAGE or A*β*-LRP-1 interaction in this study, we highlight the necessity of further study concerning the action of adiponectin related with A*β* transporters.

Moreover, previous studies demonstrated that adiponectin negatively regulates the production of pro-inflammatory cytokines including MCP-1, IL-6 in endothelial cells by modulating the activation of NF-*κ*B.^78, 79^ Based on our results, we assume that adiponectin may regulate the expression of pro-inflammatory cytokines and inflammatory responses by inhibiting the activation of NF-*κ*B in brain endothelial cells under A*β* toxicity. In particular, we found that AdipoR1 mediates the effect of adiponectin such as the suppression of NF-*κ*B phosphorylation and the protection of tight junction protein loss.

Taken together, we assume that adiponectin may alleviate the BBB disruption in AD (1) by inhibiting apoptosis of brain endothelial cells, (2) by protecting tight junction integrity, and (3) by mediating the balance of A*β* transporters in brain endothelial cells. Furthermore, AdiopoR1 may be crucial in the inflammatory response through NF-*κ*B and the loss of tight junction protein in brain endothelial cells. Hence, we suggest that the action of adiponectin through AdipoR1 may alleviate AD pathogenesis by protecting BBB disruption.

## Materials and methods

### Animal experiments

Male 5xFAD transgenic mice (strain: B6SJL-Tg [APPSwFlLon, PS1*M146L*L286V] 6799Vas/J, 5 months) were purchased from The Jackson Laboratory (Bar Harbor, ME, USA). 5xFAD mice were detected A*β*_42_ production in brain at 2 months.^80^ Wild-type male C57BL/6 mice (25–30 g, 5 months) were provided by Koatech (Koatech, Pyeongtaek, South Korea). Animal treatment and maintenance were performed in accordance with the Animal Care Guidelines of Chonnam National University, South Korea.

### Cell culture and drug treatment

We used mouse brain endothelial cells (bEnd.3 cells). bEnd.3 cells were cultured in Dulbecco modified Eagle’s medium (Gibco, Grand Island, NY, USA) which contained 0.45% glucose, 0.37% NaHCO_3_, 4 mM glutamine, 10% FBS, 100 *μ*g/ml penicillin, and 100 *μ*g/ml streptomycin. bEnd.3 cells were grown in a humidified incubator at 37 °C with 5% CO_2_. bEnd.3 cells were pretreated with Acrp 30 (10 *μ*g/ml) (Sigma-Aldrich, St. Louis, MO, USA) for 24 h and were subsequently treated with A*β* (1–20 *μ*M) for 24 h.

### A*β* oligomer preparation

Oligomeric A*β* were prepared following previous report.^81^ Synthetic A*β* 42 peptide (American Peptide, Sunnyvale, CA, USA) was dissolved to 1 mM hexafluoroisopropanol (Sigma-Aldrich). The solution was evaporated for 2 h and subsequently peptide film was resuspended in dimethyl sulfoxide (DMSO, Sigma-Aldrich) to produce a 1 mM solution. Later, to prepare oligomeric A*β* 42, this solution was diluted to 100 *μ*M in DMSO medium (Gibco) and incubated for 12 h at 4 °C.

### Preparation of the AdipoR1 and AdipoR2 targeting siRNA

A small interfering RNA for AdipoR1 (5 *μ*M) was prepared to silence AdipoR1 siRNA sc-60123 (Santa Cruz Biotechnology, Santa Cruz, CA, USA). A small interfering RNA for AdipoR2 (5 *μ*M) was prepared to silence AdipoR2 siRNA sc-46756 (Santa Cruz Biotechnology). For the transfection of siRNA, a 5 *μ*M final concentration of siRNA AdipoR1 and siAdipoR2 were mixed with lipofectamine 2000 (Invitrogen, Carlsbad, CA, USA) in Opti-MEM medium and incubated at room temperature for 15 min. The mixture was added to bEnd.3 cells in six-well plates. After 2 days, they were harvested for total protein or RNA extraction.

### 3-(4,5-dimethylthiazol-2-yl)-2,5-diphenyltetrazolium bromide (MTT) assay

bEnd.3 cells (2 × 10^5^ cells/ml) were seeded in 96-well plates to check all conditions, including Acrp30 (10 *μ*g/ml) pre-treatment (24 h) and A*β* (20 *μ*M) treatment (24 h). Later, cells were rinsed twice with PBS, and culture medium was replaced with serum-free medium. Then, 100 *μ*l of 3-(4,5-dimethylthiazol-2-yl)-2,5-diphenyltetrazolium bromide (MTT) (Sigma-Aldrich) solution (2 mg/ml in PBS) was added per well.^82^ After 90 min of incubation, medium was removed, and DMSO was added to solubilize the purple formazan product of the MTT reaction. The supernatant was measured using an ELISA reader at a wavelength of 570 nm. All experiments were repeated four times. Cell viabilities are expressed relative to non-treatment controls (considered to be 100%).^82^

### Determination of nitrite

bEnd.3 cells were plated onto 96-well plates and pre-treated with Acrp30 (10 *μ*g/ml) 24 h prior to stimulation with 20 *μ*M of A*β*. Supernatants were collected and checked nitric oxide (NO) production using Griess reagent. The Griess reagents (100 *μ*l) were added to the plate and incubated for 30 min at room temperature. The absorbance of supernatants was measured at 540 nm using the ELISA reader (Versamax Molecular Devices, Hampton, NH, USA).

### ROS assay

Oxidized DCF (reflecting the levels of H_2_O_2_ and ONOO^−^) in bEnd.3 cells was measured by using the DCF-DA (Sigma-Aldrich) assay as described previously.^83^ Later, bEnd.3 cells were washed with PBS. Then bEnd.3 cells were loaded with the probe DCF-DA (5 *μ*M) and incubated for 40 min at 37 °C in PBS. Incubated bEnd.3 cells were washed with PBS to remove the excess DCF probe. DCF images in cells were acquired by confocal microscope (Carl Zeiss, Thornwood, NY, USA) at an excitation of 488 nm and emission of 525 nm in cells.

### Hoechst 33258 and propidium iodide (PI) staining

Cell death was assessed by staining bEnd.3 cells with Hoechst 33258 dye (Sigma-Aldrich) and propidium iodide (PI; Sigma-Aldrich). Hoechst dye was added to the culture medium (10 *μ*g/ml) and samples were then incubated at 37.5 °C for 30 min. PI solution was then added (5 *μ*g/ml) just before cells were observed by confocal microscope (Carl Zeiss). PI-positive cells were counted as dead bEnd.3 cells.

### ELISA assay

bEnd.3 cells were plated in six-well plates (5 × 10^5^ cells/ml) and incubated with Acrp30 (10 *μ*g/ml) in the presence of A*β* 20 *μ*M for 24 h. The production of TNF-*α* was measured by Mouse TNF-*α* ELISA kit (eBioscience, San Diego, CA, USA; Cat No 88-7324) following the manufacturer’s instructions. The absorbance at 450 nm was detected using an ELISA microplate reader.

### Western blot analysis

bEnd.3 cells were washed with PBS and collected. Cell pellets were lysed with ice-cold RIPA buffer (Sigma-Aldrich). The lysates were centrifuged at 13 000 rpm for 30 min at 4 °C to produce whole-cell extracts. Protein (30 *μ*g) in cells was separated on a 12% SDS-polyacrylamide gel and transferred onto a polyvinylidene difluoride membrane. After blocking with skimmed milk prepared in Tris-buffered saline-tween (TBST) (20 nM Tris (pH 7.2), 150 mM NaCl, 0.1% Tween 20) for 1 h at room temperature, immunoblots were incubated for 16 h at 4 °C with primary antibodies that detect p-NF-*κ*B (1:1000, Cell Signaling, Danvers, MA, USA), NF-*κ*B (1:1000, Cell Signaling), AdipoR1 (1:1000, Abcam, Cambridge, MA, USA), AdipoR2 (1:1000, Abcam), Claudin 5 (1:1000, Cell Signaling), CD31 (1:1000, Abcam) or *β*-actin (1:1000; Millipore, Billerica, MA, USA). Blots were then incubated with each secondary antibody (Abcam) for 1 h and 30 min at room temperature. Blots were visualized by ECL solution (Millipore).

### Quantitative real-time PCR

To examine the amount of Claudin 5, ZO-1, TNF-*α*, IL-6, MCP-1, Bax, AdipoR1, AdipoR2, iNOS, eNOS, LRP-1, RAGE mRNA in bEnd.3 cells, quantitative real-time PCR was performed using each primer. Total cellular RNA was extracted from bEnd.3 cells using Trizol reagent (Invitrogen) following manual. RNA was mixed with One Step SYBR Prime Script TM RT-PCR Kit II (Takara, Otsu, Shiga, Japan) and specific primers in a total reaction volume of 20 *μ*l. PCR was performed using the following primers (5′ to 3′); Claudin 5 (F): CTG CTG GTT CGC CAA CAT T, (R): TGC GAC ACG GGC ACA G, ZO-1 (F): CAG CCG GTC ACG ATC TCC T,(R): TCC GGA GAC TGC CAT TGC, AdipoR1 (F):CCCACCATGCACTTTACTAT, (R) CACCATAGAAGTGGACGAAA, AdipoR2 (F): CAACCTTGCTTCATCTACCT, (R): CTAGCCATAAGCATTAGCCA, iNOS (F): GGG AAT CTT GGA GCG AGT TG, (R): GTG AGG GCT TGG CTG AGT GA, eNOS (F): TCCGGAAGGCGTTTGATC, (R): GCCAAATGTGCTGGTCACC; TNF-*α* (F): CGT CAG CCG ATT TGC TAT CT, (R): CGG ACT CCG CAA AGT CTA AG ; IL-6 (F): GTT GCC TTC TTG GGA CTG AT, (R): CTG GCT TTG TCT TTC TTG TTA T ; MCP-1 (F): CCC ACT CAC CTG CTG CTA CT, (R): TCT GGA CCC ATT CCT TCT TG ; Bax (F): AAG AAG CTG AGC GAG TGT, (R): GGA GGA AGT CCA ATG TC, LRP-1 (F):GAG TGT TCC GTG TAT GGC AC, (R):GAT GCC TTG GAT GAT GGT C, RAGE (F):TGG AAC CGT AAC CCT DAC CT, (R):CGA TGA TGC TGA TGC TGA CA, GAPDH (F): GAC AAG CTT CCC GTT CTC AG, (R): GAG TCA ACG GAT TTG GTC GT. Amplification cycles were conducted at 52 °C for 5 min, 95 °C for 10 s, 95 °C for 5 s, 60 °C for 35 s, and 65 °C for 15 s. Quantitative SYBR Green real-time PCR was performed with Takara PCR System (Takara) and analyzed with comparative Ct quantification. GAPDH was used as an internal control. The ΔCt values of treated cells were compared with those of untreated cells.^84^

### Reverse transcription PCR

RNA in bEnd.3 cells was isolated using Trizol Reagent (Gibco) following the manufacturer’s instructions. RT-PCR reaction was performed by using Invitrogen One step III Reverse Transcription PCR kit (Invitrogen). cDNA synthesis from mRNA and sample normalization were performed. PCR was performed using the following thermal cycling conditions: 95 °C for 10 min; 40 cycles of denaturing at 95 °C for 15 s, annealing at 58 °C for 30 s, elongation at 72 °C for 30 s; final extension at 72 °C for 5 min; and holding at 4 °C. PCR was performed using the following primers (5′ to 3′); Bcl2 (F): TACCGTCGTGACTTCGCAGAG, (R): GGCAGGCTGAGCAGGGTCTT, GAPDH (F): GAC AAG CTT CCC GTT CTC AG, (R): GAG TCA ACG GAT TTG GTC GT. PCR products were electrophoresed in 1% agarose gels and stained with cyber green. Each sample was normalized with GAPDH.

### Immunocytochemistry

bEnd.3 cells were washed thrice with PBS, and were permeabilized for 20 min. bEnd.3 cells were incubated with the primary antibodies for 16 h at 4 °C. The following primary antibodies were used: anti-rabbit AdipoR1 (1:500, Abcam), anti-goat AdipoR2 (1:500, Abcam), anti-rabbit NF-*κ*B (1:500, Cell Signaling), anti-rabbit p-NF-*κ*B (1:500, Cell Signaling), anti-rabbit CD31 (1:500, Abcam), and anti-rabbit Claudin 5 (1:500, Cell Signaling). After 16 h incubation, bEnd.3 cells were washed twice with PBS. bEnd.3 cells were incubated with each specific secondary antibody for 1 h and 30 min at room temperature. bEnd.3 cells were counterstained with 1 *μ*g/ml 4', 6-diamidino-2-phenylindole (DAPI, 1:100, Invitrogen) for 10 min at room temperature. Images were obtained using confocal microscope (Carl Zeiss).

### Immunohistochemistry

Brain sections were cut (20 *μ*m) onto coated glass slides (Thermo Scientific, Waltham, MA, USA), and fixed in acetone for 20 min at −20 °C. The slides were first washed in TBS and then incubated with methanol. To block nonspecific labeling, sections were incubated in 5% bovine serum albumin (Sigma-Aldrich) diluted in PBS for 1 h before incubation with primary and secondary antibodies. Primary antibodies for AdipoR1 (1:500, Abcam), AdipoR2 (1:500, Abcam), postsynaptic density protein 95 (PSD95) (1:500, Cell Signaling) were applied to the samples for 16 h at 4 °C, followed by 1 h incubation with appropriate florescence secondary antibody (1:500, Invitrogen) and three times washes in PBS for 5 min each. After three washes in 0.1 % PBS with Tween-20, the sections were incubated with each secondary antibody for 1 h in the dark at room temperature. Later, all sections were incubated with 1 *μ*g/ml DAPI (Sigma-Aldrich) for a counter staining. Brain tissues were then visualized under a confocal microscope (Carl Zeiss, Oberkochen, Germany).

### Statistical analysis

Statistical analysis was conducted by SPSS 18.0 software (IBM Corp., Armonk, NY, USA). Results are expressed as the mean±standard deviations (S.D). Statistical analyses were performed using one-way analysis of variance followed by Bonferroni *post-hoc* multiple comparison. Differences were considered statistically significant at *P<0.05 and **P<0.001.

## Publisher’s Note

Springer Nature remains neutral with regard to jurisdictional claims in published maps and institutional affiliations.
